# Rate of decline in residual kidney function pre and post peritoneal dialysis initiation: A *post hoc* analysis of the IDEAL study

**DOI:** 10.1371/journal.pone.0242254

**Published:** 2020-11-16

**Authors:** Isabelle Ethier, Yeoungjee Cho, Carmel Hawley, Elaine M. Pascoe, Andrea K. Viecelli, Scott B. Campbell, Carolyn van Eps, Nicole M. Isbel, Bruce A. Cooper, David C. Harris, Carol A. Pollock, Muh Geot Wong, David W. Johnson

**Affiliations:** 1 Division of Nephrology, Centre Hospitalier de l’Université de Montréal, Montréal, Canada; 2 Department of Nephrology, Princess Alexandra Hospital, Brisbane, Australia; 3 Australasian Kidney Trials Network, University of Queensland, Brisbane, Australia; 4 Translational Research Institute, Brisbane, Australia; 5 School of Medicine, University of Queensland, Brisbane, Australia; 6 Department of Renal Medicine, Royal North Shore Hospital, St Leonards, Australia; 7 Centre for Transplantation and Renal Research, Westmead Institute for Medical Research, Sydney, Australia; 8 Sydney Medical School, University of Sydney, Sydney, Australia; 9 The George Institute for Global Health, Newtown, Australia; Universidade Estadual Paulista Julio de Mesquita Filho, BRAZIL

## Abstract

**Background:**

Residual kidney function (RKF) is associated with improved survival and quality of life in dialysis patients. Previous studies have suggested that initiation of peritoneal dialysis (PD) may slow RKF decline compared to the pre-dialysis period. We sought to evaluate the association between PD initiation and RKF decline in the Initiating Dialysis Early And Late (IDEAL) trial.

**Methods:**

In this *post hoc* analysis of the IDEAL randomized controlled trial, PD participants were included if results from 24-hour urine collections had been recorded within 30 days of dialysis initiation, and at least one value pre- and one value post-dialysis commencement were available. The primary outcome was slope of RKF decline, calculated as mean of urinary creatinine and urea clearances. Secondary outcomes included slope of urine volume decline and time from PD initiation to anuria.

**Results:**

The study included 151 participants (79 early start, 72 late start). The slope of RKF decline was slower after PD initiation (-2.69±0.18mL/min/1.73m^2^/yr) compared to before PD (-4.09±0.33mL/min/1.73m^2^/yr; change in slope +1.19 mL/min/1.73m^2^/yr, 95%CI 0.48–1.90, p<0.001). In contrast, urine volume decline was faster after PD commencement (-0.74±0.05 L/yr) compared to beforehand (-0.57±0.06L/yr; change in slope -0.18L/yr, 95%CI -0.34—-0.01, p = 0.04). No differences were observed between the early- and late-start groups with respect to RKF decline, urine volume decline or time to anuria.

**Conclusions:**

Initiation of PD was associated with a slower decline of RKF compared to the pre-dialysis period.

## Introduction

Residual kidney function (RKF) is associated with improved survival and quality of life in people on dialysis [1–3]. Many studies have shown that RKF is better preserved on peritoneal dialysis (PD) than on hemodialysis [4–7]. In a small retrospective observational study of 14 patients on PD, Berlanga *et al*. showed a slower rate of RKF decline during PD than during the pre-dialysis period [8]. Similar results were found in a larger cohort of 77 new PD patients by He *et al*. [9]. Results from the Netherlands Cooperative Study on the Adequacy of Dialysis (NECOSAD) also described an attenuation in RKF decline from the period before the start of dialysis compared to 2 to 4 months after dialysis initiation [10]. In both studies previously mentioned, [9, 10] greater RKF at dialysis initiation was also an independent risk factor for RKF decline during the dialysis phase.

The Initiating Dialysis Early And Late (IDEAL) study [11], reported in 2010, randomized 828 dialysis patients to either early-start (glomerular filtration rate [GFR] between 10 and 14 mL/min/1.73m^2^) or late-start (GFR<7 mL/min/1.73m^2^ or when clinical indicators of uremia supervened) dialysis. This study, which included 366 PD patients, showed that planned early commencement of dialysis was not associated with either a survival or quality of life benefit compared to late initiation.

The aim of this *post hoc* analysis of the IDEAL study was to compare the trend of RKF in the pre- and post-PD commencement periods and to further evaluate the impact of early vs. late initiation of PD on the decline of RKF. We hypothesized that initiating patients on PD favorably alters the rate of decline of RKF.

## Methods

The present study is a *post hoc* analysis of the IDEAL trial (Australian and New Zealand Clinical Trials Registry number 12609000266268) and was approved by the ethics committee at each participating center. The detailed IDEAL study design, methodology and results have been published previously [11, 12]. The rate of decline in residual kidney function and its independent effect on morbidity and mortality were secondary outcomes of the IDEAL study. However, this study is a *post hoc* analysis restricted to PD patients included in the IDEAL study examining the change in rate of decline before and after dialysis initiation.

### Participants and data collection

For the IDEAL study, adult patients with progressive chronic kidney failure with an estimated glomerular filtration rate (GFR; using the Cockcroft-Gault equation) between 10 and 20 mL/min/1.73m^2^ were recruited between 1 July 2000 and 14 November 2006. Baseline characteristics were collected at enrollment and patients were subsequently evaluated on a 3-monthly basis, including a timed 24-hour urine collection, for a follow-up period of 3 years from enrolment, until 14 November 2009. Once GFR was first recorded to be ≤15 mL/min/1.73m^2^, patients were randomized to either commence dialysis immediately (“early-start”) or continue routine care until dialysis initiation at GFR of 5–7 mL/min/1.73m^2^ (“late-start”). Only PD patients from the original IDEAL study were evaluated for inclusion in this *post hoc* analysis. PD patients were included if results from 24-hour urine collections had been recorded within 30 days of dialysis initiation (-30 to + 30 days from start), and at least one value pre- and one value post-dialysis commencement were available. All available 24-hour urine collection results recorded during the IDEAL study, both pre- and post-dialysis start, were reviewed as part of the present analysis.

### Study outcomes

The primary outcome was the slope of decline in GFR over time, calculated from the mean of creatinine and urea clearances from a 24-hour urine collection, corrected for body surface area (BSA). BSA was calculated from weight and height of patients recorded at enrollment using the DuBois formula [13]. Patients were considered anuric at the first of two consecutive time points of patient-reported anuria or urine production of <100 mL in a 24-hour collection. Once considered anuric, GFR was set at 0 mL/min/1.73m^2^ and no subsequent value for the patient was included in analysis. Secondary outcomes included slope of decline in 24-hour urine volume over time and time from PD initiation to anuria.

### Statistical analyses

Continuous normally distributed variables were expressed as mean ± standard deviation and compared between groups (early-start vs late-start) with Student’s t-test. Continuous non-normally distributed variables were expressed as median (interquartile range) and compared using the Mann-Whitney test. Categorical variables were presented as frequencies (percentages) and compared using the chi-squared test.

For the primary outcome of slope of decline in RKF over time, a mixed-effects linear model was used with GFR as the outcome. Time (pre- vs post-initiation) and treatment group (early- vs late-start) were included as fixed effect covariates. Patient identification number was used as a random intercept to account for correlated data due to repeated measurements over time. To allow for patient-specific change over time, the time covariate was also fitted as a random effect. The main model was therefore a mixed-effects linear model with a random intercept and a random slope. An exploratory model including additional relevant patients’ baseline characteristics (age, sex, ethnicity [Caucasian vs non Caucasian], initial dialysis dose [incremental vs full], presence of diabetes and history of cardiovascular disease [composite of ischemic heart disease, congestive heart failure or peripheral vascular disease]) was also evaluated. Incremental dialysis was defined as a dialysis dose of less than 8 liters per day in PD patients at initiation. Body mass index (BMI) was not included in the models as GFR was corrected for BSA. GFR was assumed to be normally distributed and to decline in a linear pattern. This mixed model assumed missing at random patterns to accommodate missed time points. However, patients becoming non contributors once they were reported as anuric were not at random and the results should therefore be interpreted in light of other analyses, including time to anuria. The mixed-effects linear model assumed that the random intercept and the random slope had a bivariate normal distribution with a zero mean and covariance, implying that the univariate distributions of the intercept and slope were also normal. Additionally, the random error was assumed to be normally distributed with a zero mean. Those assumptions were assessed visually using distribution plots and q-q plots. The likelihood-ratio test was used for model selection, showing the random effects model to be superior to a fixed effect model.

To allow for a change in the slope at dialysis commencement, a piecewise regression model [14], in which a “breakdummy” was used to identify values greater than the structural break value, was fitted into the main mixed model. Start of dialysis was used as the break point where time was identified as 0. For patients in whom data at start of dialysis were recorded in the 30 days prior to or after dialysis commencement (-30 to +30 days), this time value was relabeled as 0. This final model estimated the rate of GFR decline per year in the pre-dialysis start period as the coefficient for time (ß_1_) and the change in slope from the preceding interval (ß_2_), which enabled testing of whether the change in slope was significant. The slope in the post-dialysis initiation period was calculated as the sum of the slope in the pre-dialysis start period and the change in slope (ß_1_+ß_2_). This piecewise regression model assumed that the intercept was the same immediately before and after the break value, in this case, at dialysis commencement. As availability of a value at dialysis start was a requirement to include patients in this *post hoc* analysis, this assumption by the model was respected.

To compare the trends of GFR over time between treatment groups (early-start or late-start), treatment by time interaction terms were evaluated. As sensitivity analyses, the change in slopes prior to, and after dialysis initiation were also assessed separately for each treatment group.

For the secondary outcome of slope of decline in 24-hour urine volume, the same analyses were used as those for the primary outcome. BMI was included in the exploratory model as a continuous variable. Time to event analysis (time to anuria) was performed by univariate Kaplan-Meier survival analysis and multivariable Cox proportional hazards model analyses.

All analyses were performed based on the intention-to-treat principle, as modality switches during follow-up were ignored. The statistical analyses were performed using Stata (version 15.1; StataCorp LLC, Texas, USA). P<0.05 was considered statistically significant.

## Results

### Population characteristics

**Fig 1:**
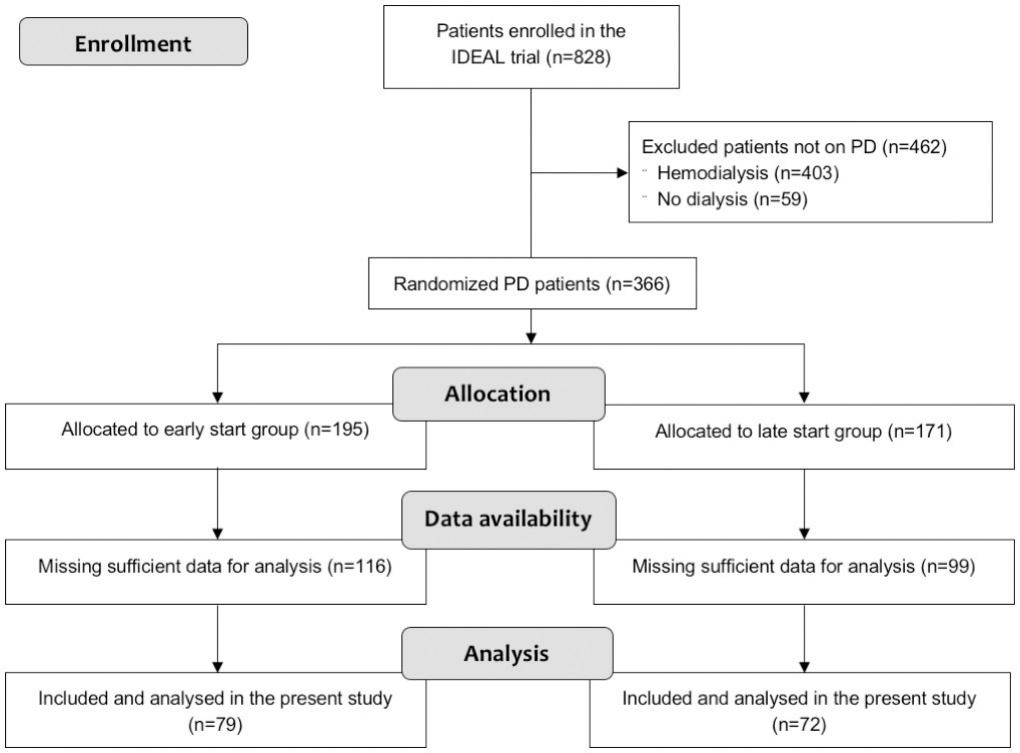
Study flow diagram. PD = peritoneal dialysis.

Of the 828 patients initially enrolled in the IDEAL trial, 366 patients were started on PD, of whom 195 and 171 respectively were allocated to the early-start and late-start groups. After exclusion of 215 patients for whom available urine collection data were insufficient for the planned analysis, 79 early-start and 72 late-start PD patients were included in this *post hoc* analysis (Fig 1). After exclusion of 5 implausibly high GFR values >30 mL/min/1.73m^2^/yr in 4 patients, 1208 values were included in analyses. For each patient, 3 to 15 GFR values were available (median = 8).

Baseline characteristics of the included patients at enrollment are presented in Table 1. Characteristics were similar between groups, except for more frequent diuretic use and a higher mean BMI in the early-start group (although distribution of BMI categories was equivalent to that of the late-start group). Diuretic use was not included in the model as it was only evaluated at enrollment and not recorded at dialysis commencement in the IDEAL study.

**Table 1:** Baseline characteristics of the included patients at enrollment.

	EARLY	LATE	*P* value	TOTAL
	n = 79	n = 72	early vs late	n = 151
**Age (years)**	62.8 ± 11.6	60.7 ± 10.9	0.2	61.8 ± 11.3
**Male sex**	49 (62%)	51 (71%)	0.3	100 (66%)
**Ethnicity**			0.8	
Caucasian	57 (72%)	53 (74%)		110 (73%)
Non-Caucasian	22 (28%)	19 (26%)		41 (27%)
**Height (m)**	1.67 ± 0.09	1.69 ± 0.09	0.1	1.68 ± 0.09
**Weight (kg)**	78.6 ± 13.5	76.6 ± 14.6	0.4	77.7 ± 14.0
**BMI (kg/m**^**2**^**)**	28.3 ± 4.6	26.7 ± 4.0	0.02*	27.6 ± 4.4
<18.5	0 (0%)	1 (1%)		1 (1%)
18.5–24.9	20 (25%)	23 (32%)		43 (28%)
25–29.9	34 (43%)	33 (46%)		67 (44%)
30+	25 (32%)	15 (21%)		40 (26%)
**Primary kidney disease**			0.9	
Diabetic nephropathy	27 (34%)	24 (33%)		51 (34%)
Hypertension/ Renovascular disease	13 (16%)	11 (15%)		24 (16%)
Glomerulonephritis	11 (14%)	10 (14%)		21 (14%)
Polycystic kidney disease	5 (6%)	8 (11%)		13 (9%)
Other	23 (29%)	19 (26%)		42 (28%)
**Failed transplant**	1 (1%)	2 (3%)	0.5	3 (2%)
**PD modality**			0.6	
CAPD	76 (96%)	68 (94%)		144 (95%)
APD	3 (4%)	4 (6%)		7 (5%)
**Initial dialysis dose**			0.8	
Incremental	11 (14%)	9 (13%)		20 (13%)
Full	68 (86%)	63 (88%)		131 (87%)
**Smoking status**			0.9	
Never	31 (39%)	27 (38%)		58 (38%)
Current	5 (6%)	5 (7%)		10 (7%)
Former	43 (54%)	40 (56%)		83 (55%)
**Comorbidities**				
Diabetes mellitus	35 (44%)	28 (39%)	0.5	63 (42%)
Hypertension	72 (91%)	67 (93%)	0.7	139 (92%)
CVD	30 (38%)	26 (36%)	0.8	56 (37%)
**Medication**				
Diuretic	47 (59%)	29 (40%)	0.02	76 (50%)
ACE inhibitor/Angiotensin II blocker	50 (63%)	50 (69%)	0.4	100 (66%)

ACE = angiotensin-converting enzyme; APD = automated peritoneal dialysis; BMI = body mass index; CAPD = continuous ambulatory peritoneal dialysis; CVD = cardiovascular disease (defined as a composite of ischemic heart disease, congestive heart failure and peripheral vascular disease); PD = peritoneal dialysis.

Values are expressed as frequency (percentage) for categorical variables, mean ± standard deviation for normally distributed continuous variables, and median (interquartile range) for non-normally distributed continuous variables.

There were no missing data for all variables reported.

*Reported *P* value for BMI as a continuous variable compared between groups. *P* value for BMI categories between groups = 0.3.

Median total observation time was 2.6 years and similar between groups. However, patients in the late-start group had longer observation time in the pre-dialysis initiation period and slightly shorter (2 months) observation time in the PD period (Table 2). Median GFR (calculated from urine collection) at start of observation time was 9.9 mL/min/1.73m^2^ and similar between groups. As expected, median GFR was significantly different between groups at dialysis initiation (7.8 [6.2–10.3] vs 6.1 [4.9–7.6] mL/min/1.73m^2^ in the early and late groups, respectively). However, urine volumes at start of observation time and at dialysis initiation were both similar between groups (Table 2). Over the study period, 49 (32%) patients became anuric (26 and 23 in the early-start and late-start groups, respectively). Anuria occurred 0.3 to 3.0 years after PD commencement (Table 2).

**Table 2:** Descriptive data on observation time, glomerular filtration rate, 24-hour urine volume and anuria.

	EARLY	LATE	*P* value	TOTAL
	n = 79	n = 72	early vs late	n = 151
**OBSERVATION TIME WITH AVAILABLE URINE COLLECTIONS**				
**Total observation time (in years)**	2.5 (1.2–3.1)	2.7 (1.9–3.0)	0.3	2.6 (1.5–3.0)
**Observation time pre dialysis initiation (in years)**	0.3 (0.1–0.8)	0.9 (0.5–1.6)	<0.001	0.6 (0.2–1.2)
**Observation time post dialysis initiation (in years)**	1.7 (0.8–2.7)	1.5 (0.8–1.9)	0.05	1.6 (0.8–2.2)
**GFR**				
**GFR at start of observation time (mL/min/1.73m**^**2**^**)**	10.3 (8.1–13.0)	9.9 (7.9–12.7)	0.6	9.9 (8.0–12.9)
**GFR at dialysis initiation (mL/min/1.73m**^**2**^**)**	7.8 (6.2–10.3)	6.1 (4.9–7.6)	<0.001	6.7 (5.5–9.4)
**24-HOUR URINE VOLUME**				
**Urine volume at start of observation time (in L)**	1.95 (1.63–2.53)	2.06 (1.70–2.72)	0.3	1.98 (1.66–2.63)
**Urine volume at dialysis initiation (in L)**	1.75 (1.36–2.14)	1.77 (1.26–2.22)	0.8	1.75 (1.30–2.18)
**ANURIA**				
**Number of patients who became anuric (%)**	26 (33%)	23 (32%)	0.9	49 (32%)
**Time to anuria from start of observation time (in years)**	2.5 (1.4–3.0)	2.5 (1.7–2.7)	0.9	2.5 (1.5–2.7)
**Time to anuria from dialysis initiation (in years)**	1.7 (1.2–2.3)	1.7 (1.0–2.2)	0.5	1.7 (1.2–2.2)

GFR = glomerular filtration rate, calculated as mean of creatinine and urea clearances from a 24-hour urine collection, corrected for body surface area.

Values are expressed as median (interquartile range) if not indicated otherwise.

### Primary outcome

**Fig 2:**
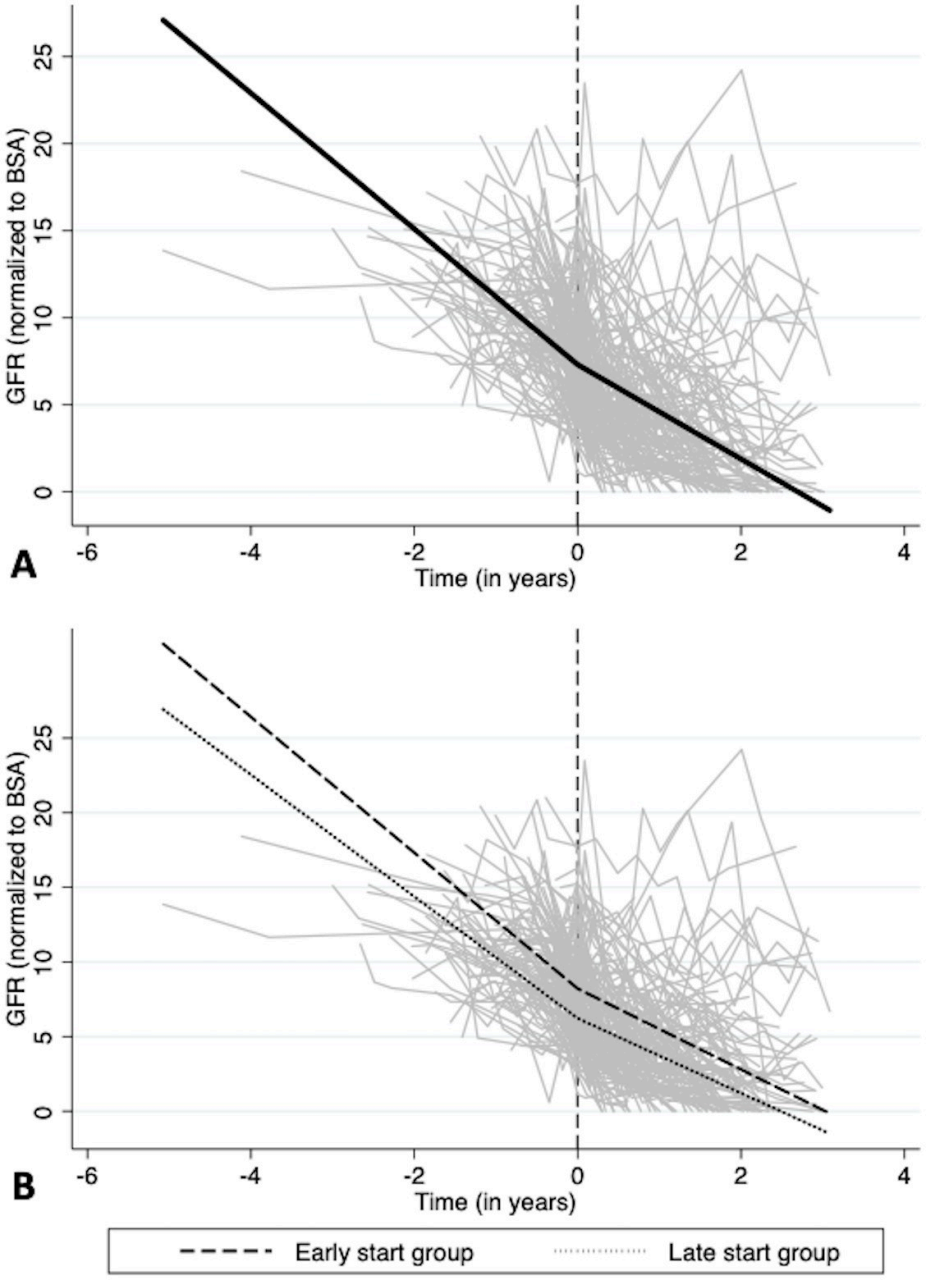
Trend of glomerular filtration rate (normalized to BSA) over time [A] in all patients and [B] for each treatment group. The gray lines represent individual patient measurements and the black lines represent the predicted slopes in the pre- and post-dialysis initiation periods.

The overall decline in GFR over the study period was -2.99±0.16 mL/min/1.73m^2^/yr. When allowing for the change in slope at dialysis commencement, the slope of GFR decline was slower following PD commencement (-2.71±0.18 mL/min/1.73m^2^/yr) compared to the pre-dialysis period (-3.90±0.32 mL/min/1.73m^2^/yr; change in slope +1.19 mL/min/1.73m^2^/yr, 95% CI 0.48–1.90, p = 0.001) (Table 3; Fig 2A). Following adjustment for treatment group, the slope of GFR decline was still slower following PD commencement (-2.69±0.18 mL/min/1.73m^2^/yr) compared to the pre-dialysis period (-4.09±0.33 mL/min/1.73m^2^/yr; change in slope +1.19 mL/min/1.73m^2^/yr, 95% CI 0.48–1.90, p<0.001). Almost identical results were observed after further adjustment for baseline patient characteristics (Table 3).

**Table 3:** Trend of glomerular filtration rate over time (in mL/min/1.73m^2^/yr) for all patients.

	ALL PATIENTS			
	n = 151			
Overall trend over time	-2.99 ± 0.16			
Trend during the pre- and post-dialysis initiation periods	PRE	POST	CHANGE	
			Value (95% CI)	*P* value
Unadjusted model	-3.90 ± 0.32	-2.71 ± 0.18	+1.19 (0.48–1.90)	0.001
Adjusted for treatment group model	-4.09 ± 0.33	-2.69 ± 0.18	+1.40 (0.68–2.12)	<0.001
Exploratory model*	-4.10 ± 0.33	-2.69 ± 0.18	+1.41 (0.67–2.14)	<0.001

*Adjusted for patients’ characteristics at enrollment: treatment group (early- vs. late-start), age, sex, ethnicity (Caucasian vs non-Caucasian), initial dialysis dose (incremental vs full), presence of diabetes mellitus and history of cardiovascular disease.

No statistically significant interaction was found between time and treatment group (early-start versus late-start). In a sensitivity analysis looking at trends of GFR over time separately in each group, slopes were -4.55±0.68 and -2.71±0.28 mL/min/1.73m^2^/yr in the pre- and post-dialysis periods (change in slope +1.84 (0.43–3.25); p<0.001) in the early-start group and -4.08±0.39 and -2.50±0.19 mL/min/1.73m^2^/yr (change in slope +1.58 (0.69–2.46); p<0.001) in the late-start group (Table 4; Fig 2B; S1 Fig). Adjustment for baseline patient characteristics resulted in similar values.

**Table 4:** Trend of glomerular filtration rate over time (in mL/min/1.73m^2^/yr) in the early and late dialysis start groups.

	EARLY-START GROUP				LATE-START GROUP			
	n = 79				n = 72			
Overall trend over time	-2.93 ± 0.26				-3.05 ± 0.17			
Trend during the pre- and post-dialysis initiation periods	PRE	POST	CHANGE		PRE	POST	CHANGE	
			Value (95% CI)	*P* value			Value (95% CI)	*P* value
Unadjusted model	-4.55 ± 0.68	-2.71 ± 0.28	+1.84 (0.43–3.25)	<0.001	-4.08 ± 0.39	-2.50 ± 0.19	+1.58 (0.69–2.46)	<0.001
Exploratory model*	-4.68 ± 0.71	-2.71 ± 0.28	+1.97 (0.50–3.44)	0.008	-4.07 ± 0.39	-2.49 ± 0.19	+1.58 (0.70–2.46)	<0.001

*Adjusted for patients’ characteristics at enrollment: age, sex, ethnicity (Caucasian vs non-Caucasian), initial dialysis dose (incremental vs full), presence of diabetes mellitus and history of cardiovascular disease.

### Secondary outcomes

The overall decline in 24-hour urine volume over the study period was -0.64±0.03 L/yr. When allowing for the change in slope at dialysis commencement, urine volume decline was faster after PD commencement (-0.74±0.05 L/yr) than in the pre-dialysis period (-0.57±0.06 L/yr; change in slope -0.18 L/yr, 95% CI -0.34—-0.01, p = 0.04) (S1 Table; S2A Fig). Adjusting for treatment group and further adjusting for other patients’ baseline characteristics produced similar results (S1 Table).

The effect of group allocation in the IDEAL study was explored by including an interaction term in the model which showed that the between-group differences in slopes and pre/post changes in urine volume were also not statistically significant. Despite the non-significant results for the effect of group allocation, sub-group analyses were done. Change in the slopes of urine volume over time in the pre- and post-dialysis initiation periods was not statistically significant in the early-start group (difference +0.01 L/yr, 95% CI -0.29 to 0.31, p = 0.96), whereas the rate of decline in urine volume during the PD period was higher than in the pre-dialysis period in the late-start group (difference -0.26 L/yr, 95% CI -0.49 to -0.04, p = 0.02) (S2 Table and S2B and S2C Fig). Adjustment for patients’ baseline characteristics yielded similar values.

Time to anuria from PD start was similar between the early-start and late-start groups (log-rank test p-value = 0.08; S3 Fig). Using multivariable Cox regression including variables with a p-value <0.2 at the univariable level, higher BMI and incremental initial dialysis dose were associated with shorter time to anuria (S3 Table). However, it should be noted that only 20 patients overall (11 early-start and 9 late-start) were started on an incremental regimen. Of those, 11 (8 early-start and 3 late-start) became anuric over the study period. Treatment group was not associated with time to anuria from PD start.

Additional analyses were also performed to evaluate time to anuria from start of observation time, at which time point GFR was similar between groups (Table 2). Kaplan-Meier survival curves were comparable between groups (log-rank test p-value = 0.62; S4 Fig) and treatment group was not significantly associated with time to anuria from start of observation time in univariable Cox regression. In multivariable Cox regression, higher BMI was associated with a shorter time to anuria (S3 Table).

## Discussion

This *post hoc* analysis of the IDEAL trial, evaluating the early and late initiation of dialysis, showed that PD initiation was associated with a significantly slower decline of RKF, defined as GFR normalized to BSA, compared to pre-dialysis. This attenuation in the rate of decline was not significantly different between the early and late PD start groups. Time to anuria was also similar irrespective of the timing of dialysis commencement.

Results from this study on the association of PD initiation with GFR trend are in line with previous reports in PD patients from the NECOSAD study [10] and two other retrospective studies [8, 9]. However, the rate of decline reported in the present study in the pre-dialysis period was much lower (3.9 mL/min/1.73m^2^/yr) than those reported in the three previously mentioned studies (6.6 [10], 7.1 [9] mL/min/1.73m^2^/yr and 11.3 [8] mL/min/yr). These discrepancies may be explained by different methods of GFR estimation across the different studies (creatinine clearance from 24-hour urinary and serum creatinine;[8] MDRD formula in the pre-dialysis period [9] and average of creatinine and urea clearances from 24-hour urine collection, corrected for BSA, in the present study) and by a longer pre-dialysis observation time in the present study compared to both the NECOSAD study [10] and the Canadian study [9] (only evaluated the year preceding dialysis commencement). Moreover, the pre-dialysis rate of GFR decline observed in our study is in line with the findings from a systematic review [15] of studies reporting weighted annual mean GFR decline of 2.4 mL/min/1.73m^2^/yr in chronic kidney disease cohorts (3.0 mL/min/1.73m^2^/yr in stage 5 CKD). The rate of GFR decline in the PD period observed in the present study is similar to values reported in previous studies [9, 16], although higher than that reported in the NECOSAD study (1.32 mL/min/1.73m^2^/yr) [10].

The slower decline of GFR in the PD period compared to the pre-dialysis period was observed in both the early and late PD start groups and no significant interaction was found between change in RKF slope and allocated group.

For the secondary outcome, commencement of PD was associated with a faster decline in urine volume than in the pre-PD period. This result contrasts with the findings for GFR decline, which was attenuated with PD initiation. However, we are uncertain of the significance of the findings related to urine volume because diuretic use surrounding the time of dialysis initiation could not be assessed in this study. This warrants careful interpretation of urine volume analyzed independently of measured solute clearance in this context, as supported by a previous study on the role of diuretics in the preservation of RKF in PD patients showing that urea and creatinine clearances were not affected by diuretics administration, whereas urine volume was increased with diuretic use [17]. Although no interaction was shown between allocation groups (early- vs. late-start) and the change in rate of decline in urine volume, sub-group analyses showed diverging results. This should be interpreted with caution as diuretic use at dialysis initiation could not be assessed and may have been different between the early- and the late-start group.

Furthermore, findings from the current study reinforce the assertion that urine volume is not a good surrogate for GFR and that both urine volume and GFR should be assessed in PD patients with efforts made to preserve both. This is also supported by results from the CANUSA study [18]. This study, evaluating the association of adequacy of dialysis with clinical outcomes in incident PD patients, found a correlation of total (renal and peritoneal) solute clearance with morbidity and mortality outcomes [18]. In a reanalysis of the CANUSA study, lower GFR was associated with mortality in a multivariable Cox regression model. However, when urine volume was added to the model, the association of GFR with survival was removed, while an increase of 250 ml of urine per day was associated with a 36% decrease in the risk ratio of death [19].

In the evaluation of anuria, higher BMI was consistently associated with this outcome, in keeping with the findings of previous studies [20–22]. Time to anuria from either PD commencement or start of observation time was not significantly different between early and late dialysis start groups. This finding is in line with the main conclusions from the IDEAL trial, which found similar clinical outcomes (including survival) between the two study groups. The association of PD initiation with a slowing of RKF decline should be balanced against the economic implications and impact on quality of life of PD commencement. It should also be kept in mind that RKF is a surrogate outcome and earlier initiation of PD was not shown to be associated with either a longer time to anuria in the present *post hoc* analysis or better survival in the original IDEAL study.

Many hypotheses have been evoked to explain an apparent attenuation in the rate of decline of RKF with PD initiation. PD itself might help reduce the burden on the remaining glomeruli through removal of solute and fluid in a continuous manner, thereby decreasing hyperfiltration injury and the subsequent GFR decline. Peritoneal urea and creatinine clearance in PD patients contributes to removal of those solutes, thereby decreasing their filtered glomerular load. Consequently, the estimation of glomerular filtration rate using measurements of small solute clearance might be affected by the additional peritoneal clearance. On the other hand, this peritoneal clearance might have an impact on the osmolar load of urea, which could decrease its contribution to urea-induced osmotic diuresis. Thus, the combined evaluation of both eGFR and urine volume in PD patients appears to have more value than a single measurement to assess residual kidney function. Nephrotoxic solute accumulation [23], oxidative stress [24] and metabolic acidosis [25] are also possible contributors to GFR decline in the pre-dialysis phase which might be corrected by PD, thereby mitigating their effect on RKF loss. Finally, more gentle, continuous fluid removal by PD might better preserve RKF compared to intensive diuretic bolus use with important hemodynamic changes in the pre-dialysis period.

The strengths of this study include its large sample size, long median observation period (2.6 years), involvement of 32 centers and regular, scheduled 24-hour urine volume collections which enhanced both internal and external validity. Moreover, in contrast to some of the previous studies [9], the same GFR measure was used in both the pre- and post-dialysis periods, which enabled fair comparison of RKF decline in these periods.

However, those strengths should be balanced with the limitations of the study. As this was a *post hoc* analysis of a previous randomized controlled trial, it was not designed specifically for this aim. Consequently, the observation times in both the pre- and post-dialysis periods varied widely between patients and across groups. In particular, pre-dialysis follow-up time was shorter in the early start group because, in order to mitigate the risk of lead time bias, patients were randomized when their GFR fell below 15 mL/min/1.73m^2^ to either early start (GFR 10–15 mL/min/1.73m^2^) or late start (5–7 mL/min/1.73m^2^). Since GFR at time of enrollment was comparable in both groups (10.3 (8.1–13.0) vs 9.9 (7.9–12.7) mL/min/1.73m^2^), time from enrollment to dialysis commencement was shorter in the early-start group, thereby reducing the time over which GFR slope was observed. As part of the initial study, medications were recorded at enrollment and subsequently at each follow-up visit after dialysis start. Unfortunately, they were not recorded during the pre-dialysis initiation period and were not available for the majority of patients at the time of dialysis commencement. Therefore, it was not possible to reliably account for medications in our models and use of certain relevant medications (particularly diuretics and angiotensin converting enzyme inhibitors) should be considered as a potential confounder. Moreover, other risk factors known to be associated with RKF decline were not able to be evaluated. These included, but were not limited to the use of nephrotoxic drugs and intravascular radiocontrast, serum bicarbonate levels in the pre-dialysis period, rate of peritonitis in the PD period, occurrence of episodes of dehydration and degree of proteinuria. Due to the number of patients included, it was not possible to adjust for all known patient characteristics, such as primary kidney disease. However, the most relevant factors associated with loss of RKF identified in previous studies (for example, diabetes) were included in analyses. Furthermore, dialysis dose was not included in analyses because the piecewise regression model used the pre- and post-dialysis initiation periods in the same model and the exact dialysis dose was not recorded at dialysis initiation for 87% of patients. Nevertheless, incremental vs. full dialysis dose at initiation was recorded as a binary variable, which was included in the exploratory model. Finally, analyses were based on the assumption that RKF declines in a linear fashion. The analyses conducted allowed for a clinically relevant interpretable estimation of the decline and was in line with previous studies based on the same assumption.

In conclusion, this *post hoc* analysis of the IDEAL study shows that the rate of decline in GFR is lower in the PD period compared to the pre-dialysis initiation period, which was seen in both the early-start and late-start dialysis groups. The trend in urine volume over time differed from that of GFR measurements normalized to BSA, thus supporting the evaluation of RKF based on estimation of GFR rather than urine volume alone.

## Supporting information

S1 Table Trend of 24-hour urine volume over time (in L/yr) for all patients. (DOCX)

S2 Table Trend of 24-hour urine volume over time (in L/yr) in the early and late dialysis start groups. (DOCX)

S3 Table Hazard ratio for time to anuria including significant (p<0.2) covariates in univariable regression. (DOCX)

S1 Fig Trend of glomerular filtration rate (normalized to BSA) over time for each treatment group. The gray lines represent individual patient measurements and the black lines represent the predicted slopes in the pre- and post-dialysis initiation periods.
(TIF)

S2 Fig Trend of urine volume over time [A] in all patients and [B & C] for each treatment group. The gray lines represent individual patient measurements and the black lines represent the predicted slopes in the pre- and post-dialysis initiation periods.
(TIF)

S3 Fig Time to anuria from peritoneal dialysis initiation comparing early and late dialysis start groups. (TIF)

S4 Fig Time to anuria from start of observation time comparing early and late dialysis start groups. At start of observation time, GFR was similar between groups (10.3 [8.1–13.0] vs 9.9 [7.9–12.7] mL/min/1.73m^2^ in the early and late-start group, respectively).
(TIF)

S1 Checklist CONSORT 2010 checklist of information to include when reporting a randomised trial*. (PDF)

S1 Study protocol (PDF)

## References

[1] Termorshuizen F, Korevaar JC, Dekker FW, Van Manen JG, Boeschoten EW, Krediet RT. The Relative Importance of Residual Renal Function Compared With Peritoneal Clearance for Patient Survival and Quality of Life: An Analysis of the Netherlands Cooperative Study on the Adequacy of Dialysis (NECOSAD)-2. Am J Kidney Dis. 2003;41(6):1293–302. 10.1016/s0272-6386(03)00362-7 12776283

[2] Termorshuizen F, Dekker FW, Van Manen JG, Korevaar JC, Boeschoten EW, Krediet RT. Relative Contribution of Residual Renal Function and Different Measures of Adequacy to Survival in Hemodialysis Patients: An analysis of the Netherlands Cooperative Study on the Adequacy of Dialysis (NECOSAD)-2. J Am Soc Nephrol. 2004;15(4):1061–70. 10.1097/01.asn.0000117976.29592.93 15034110

[3] Perl J, Bargman JM. The Importance of Residual Kidney Function for Patients on Dialysis: A Critical Review. Am J Kidney Dis. 2009;53(6):1068–81. 10.1053/j.ajkd.2009.02.012 19394737

[4] Lysaght MJ, Vonesh EF, Gotch F, Ibels L, Keen M, Lindholm B, et al The Influence of Dialysis Treatment Modality on the Decline of Remaining Renal Function. ASAIO Trans. 1991;37:598–604. 1768496

[5] Moist LM, Port FK, Orzol SM, Young EW, Ostbye T, Wolfe RA, et al Predictors of Loss of Residual Renal Function among New Dialysis Patients. J Am Soc Nephrol [Internet]. 2000;11(3):556–64. Available from: http://jasn.asnjournals.org/content/11/3/556.full.pdf 10703680 10.1681/ASN.V113556

[6] Lang SM, Bergner A, Töpfer M, Schiffl H. Preservation of residual renal function in dialysis patients: Effects of dialysis-technique-related factors. Perit Dial Int. 2001;21(1):52–8. 11280496

[7] Jansen MAM, Hart AAM, Korevaar JC, Dekker FW, Boeschoten EW, Krediet RT. Predictors of the rate of decline of residual renal function in incident dialysis patients. Kidney Int. 2002;62(3):1046–53. 10.1046/j.1523-1755.2002.00505.x 12164889

[8] Berlanga JR, Marron B, Reyero A, Caramelo C, Ortiz A. Peritoneal Dialysis Retardation of Progression of Advanced Renal Failure. Perit Dial Int. 2002;22(2):239–42. 11990410

[9] He L, Liu X, Li Z, Abreu Z, Malavade T, Lok CE, et al Rate of decline of residual kidney function before and after the start of peritoneal dialysis. Perit Dial Int [Internet]. 2016;36(3):334–9. Available from: http://www.pdiconnect.com/content/36/3/334.full.pdf%0Ahttp://ovidsp.ovid.com/ovidweb.cgi?T=JS&PAGE=reference&D=emed18&NEWS=N&AN=610521888 10.3747/pdi.2016.00024 PMC4881797 27044795

[10] De Jager DJ, Halbesma N, Krediet RT, Boeschoten EW, Le Cessie S, Dekker FW, et al Is the decline of renal function different before and after the start of dialysis? Nephrol Dial Transplant. 2013;28(3):698–705. 10.1093/ndt/gfs578 23300262

[11] Cooper BA, Branley P, Bulfone L, Collins JF, Craig JC, Fraenkel MB, et al A Randomized, Controlled Trial of Early versus Late Initiation of Dialysis. N Engl J Med. 2010;363(7):609–19. 10.1056/NEJMoa1000552 20581422

[12] Cooper BA, Branley P, Bulfone L, Collins JF, Craig JC, Dempster J, et al The Intiating Dialysis Early And Late (ideal) study: Study rationale and design. Perit Dial Int. 2004;24(2):176–81. 15119639

[13] Du Bois D, Du Bois EF. A formula to estimate the approximate surface area if height and weight be known. Vol. 5, Nutrition. 1989 p. 303–11. 2520314

[14] UCLA: Statistical Consulting Group. How can I run a piecewise regression in STATA? | STATA FAQ [Internet]. [cited 2020 Jan 22]. https://stats.idre.ucla.edu/stata/faq/how-can-i-run-a-piecewise-regression-in-stata

[15] Janmaat CJ, van Diepen M, van Hagen CCE, Rotmans JI, Dekker FW, Dekkers OM. Decline of kidney function during the pre-dialysis period in chronic kidney disease patients: A systematic review and meta-analysis. Clin Epidemiol. 2018;10:613–22. 10.2147/CLEP.S153367 29872350 PMC5973628

[16] Johnson DW, Brown FG, Clarke M, Boudville N, Elias TJ, Foo MWY, et al Effects of Biocompatible versus Standard Fluid on Peritoneal Dialysis Outcomes. J Am Soc Nephrol. 2012;23(6):1097–107. 10.1681/ASN.2011121201 22440906 PMC3358767

[17] Medcalf JF, Harris KPG, Walls J. Role of diuretics in the preservation of residual renal function in patients on continuous ambulatory peritoneal dialysis. Kidney Int. 2001;59(3):1128–33. 10.1046/j.1523-1755.2001.0590031128.x 11231370

[18] Churchill DN, Taylor DW, Keshaviah PR, Thorpe KE, Beecroft ML, Jindal KK, et al Adequacy of dialysis and nutrition in continuous peritoneal dialysis: Association with clinical outcomes. J Am Soc Nephrol. 1996;7(2):198–207. 8785388 10.1681/ASN.V72198

[19] Bargman JM, Thorpe KE, Churchill DN. Relative contribution of residual renal function and peritoneal clearance to adequacy of dialysis: A reanalysis of the CANUSA study. J Am Soc Nephrol. 2001;12(10):2158–62. 11562415 10.1681/ASN.V12102158

[20] Liao C Te, Shiao CC, Huang JW, Hung KY, Chuang HF, Chen YM, et al Predictors of faster decline of residual renal function in Taiwanese peritoneal dialysis patients. Perit Dial Int. 2008;28(SUPP. 3):S191–5. 18552254

[21] Singhal MK, Bhaskaran S, Vidgen E, Bargman JM, Vas SI, Oreopoulos DG. Rate of decline of residual renal function in patients on continuous peritoneal dialysis and factors affecting it. Perit Dial Int. 2000;20:429–38. 11007375

[22] Johnson DW, Mudge DW, Sturtevant JM, Hawley CM, Campbell SB, Isbel NM, et al Predictors of Decline of Residual Renal Function in New Peritoneal Dialysis Patients. Perit Dial Int. 2003;23(16):276–83. 12938830

[23] Van Den Brand JAJG, Mutsaers HAM, Van Zuilen AD, Blankestijn PJ, Van Den Broek PH, Russel FGM, et al Uremic solutes in chronic kidney disease and their role in progression. PLoS One. 2016;11(12):1–16. 10.1371/journal.pone.0168117 PMC5199014 28033375

[24] Ueda A, Nagai K, Hirayama A, Saito C, Yamagata K. Peritoneal Dialysis Preserves Residual Renal Function and Reduces Oxidative Stress During the Initial Period of Dialysis Therapy. Adv Perit Dial. 2017;33:18–21. 29668425

[25] Phisitkul S, Khanna A, Simoni J, Broglio K, Sheather S, Rajab MH, et al Amelioration of metabolic acidosis in patients with low GFR reduced kidney endothelin production and kidney injury, and better preserved GFR. Kidney Int [Internet]. 2010;77(7):617–23. Available from: 10.1038/ki.2009.519 20072112

