# Supplementary material for: Rate of decline in residual kidney function pre and post peritoneal dialysis initiation: A post hoc analysis of the IDEAL study
Source: PLoS One. 2020 Nov 16;15(11):e0242254. doi: 10.1371/journal.pone.0242254 (PMC7668577; doi:10.1371/journal.pone.0242254)
Supplement: S1 Table — (DOCX) [file pone.0242254.s001.docx]

**S1 Table. Trend of 24-hour urine volume over time (in L/yr) for all patients**

|  | **ALL PATIENTS** | | | |
| --- | --- | --- | --- | --- |
|  | n=151 | | | |
| **Overall trend over time** | -0.64 ± 0.03 | | | |
| **Trend during the pre- and post-dialysis initiation periods** | **PRE** | **POST** | **CHANGE** | |
|  |  |  | **Value (95% CI)** | ***P* value** |
| Unadjusted model | -0.57 ± 0.06 | -0.74 ± 0.05 | -0.18 (-0.34—-0.01) | 0.04 |
| Adjusted for treatment group model | -0.57 ± 0.06 | -0.74 ± 0.05 | -0.17 (-0.34—-0.01) | 0.04 |
| Exploratory model* | -0.56 ± 0.06 | -0.75 ± 0.05 | -0.19 (-0.36—-0.02) | 0.03 |

*Adjusted for patients’ characteristics at enrollment: treatment group (early- vs. late-start), age, sex, ethnicity (Caucasian vs non-Caucasian), initial dialysis dose (incremental vs full), body mass index, presence of diabetes mellitus and history of cardiovascular disease.
