# Supplementary material for: Rate of decline in residual kidney function pre and post peritoneal dialysis initiation: A post hoc analysis of the IDEAL study
Source: PLoS One. 2020 Nov 16;15(11):e0242254. doi: 10.1371/journal.pone.0242254 (PMC7668577; doi:10.1371/journal.pone.0242254)
Supplement: S2 Table — (DOCX) [file pone.0242254.s002.docx]

**S2 Table. Trend of 24-hour urine volume over time (in L/yr) in the early and late dialysis start groups.**

|  | **EARLY-START GROUP** | | | | **LATE-START GROUP** | | | |
| --- | --- | --- | --- | --- | --- | --- | --- | --- |
|  | n=79 | | | | n=72 | | | |
| **Overall trend over time** | -0.61 ± 0.05 | | | | -0.68 ± 0.04 | | | |
| **Trend during the pre- and post-dialysis initiation periods** | **PRE** | **POST** | **CHANGE** | | **PRE** | **POST** | **CHANGE** | |
|  |  |  | **Value (95% CI)** | ***P* value** |  |  | **Value (95% CI)** | ***P* value** |
| Unadjusted model | -0.67 ± 0.12 | -0.66 ± 0.06 | +0.01 (-0.29—0.31) | 0.9 | -0.56 ± 0.07 | -0.82 ± 0.07 | -0.26 (-0.49–-0.04) | 0.02 |
| Exploratory model* | -0.65 ± 0.12 | -0.67 ± 0.07 | -0.02 (-0.32—0.28) | 0.9 | -0.56 ± 0.07 | -0.84 ± 0.07 | -0.29 (-0.51–-0.06) | 0.01 |

*Adjusted for patients’ characteristics at enrollment: age, sex, ethnicity (Caucasian vs non-Caucasian), initial dialysis dose (incremental vs full), body mass index, presence of diabetes mellitus and history of cardiovascular disease.
