# Supplementary material for: Rate of decline in residual kidney function pre and post peritoneal dialysis initiation: A post hoc analysis of the IDEAL study
Source: PLoS One. 2020 Nov 16;15(11):e0242254. doi: 10.1371/journal.pone.0242254 (PMC7668577; doi:10.1371/journal.pone.0242254)
Supplement: S3 Table — (DOCX) [file pone.0242254.s003.docx]

**S3 Table. Hazard ratio for time to anuria including significant (p<0.2) covariates in univariable regression.**

|  | **TIME TO ANURIA FROM PD START** | | **TIME TO ANURIA FROM START**  **OF OBSERVATION TIME** | |
| --- | --- | --- | --- | --- |
|  | **HR (95% CI)** | ***P* value** | **HR (95% CI)** | ***P* value** |
| **Late-start group (vs. early-start group)** | 1.80 (0.98-3.28) | 0.06 |  |  |
| **Body mass index** | 1.08 (1.01-1.15) | 0.02 | 1.09 (1.02-1.17) | 0.01 |
| **Incremental dialysis start (vs. full)** | 2.87 (1.42-5.79) | 0.003 | 2.02 (1.00-4.09) | 0.05 |
| **Diabetes** | 1.47 (0.81-2.66) | 0.2 | 1.35 (0.74-2.47) | 0.3 |
| **Cardiovascular disease** | 1.30 (0.72-2.33) | 0.4 | 1.29 (0.71-2.35) | 0.4 |
