# Supplementary figures and images for: Rate of decline in residual kidney function pre and post peritoneal dialysis initiation: A post hoc analysis of the IDEAL study
Source: PLoS One. 2020 Nov 16;15(11):e0242254. doi: 10.1371/journal.pone.0242254 (PMC7668577; doi:10.1371/journal.pone.0242254)

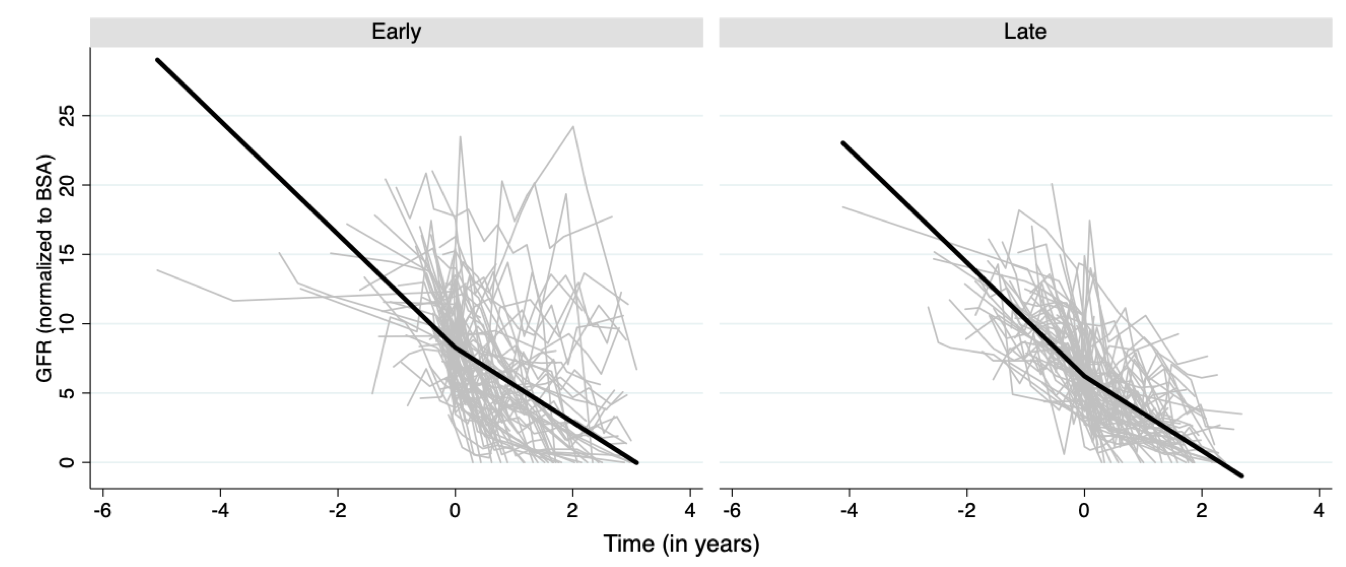

Supplement: S1 Fig — The gray lines represent individual patient measurements and the black lines represent the predicted slopes in the pre- and post-dialysis initiation periods. (TIF) [file pone.0242254.s004.tif]

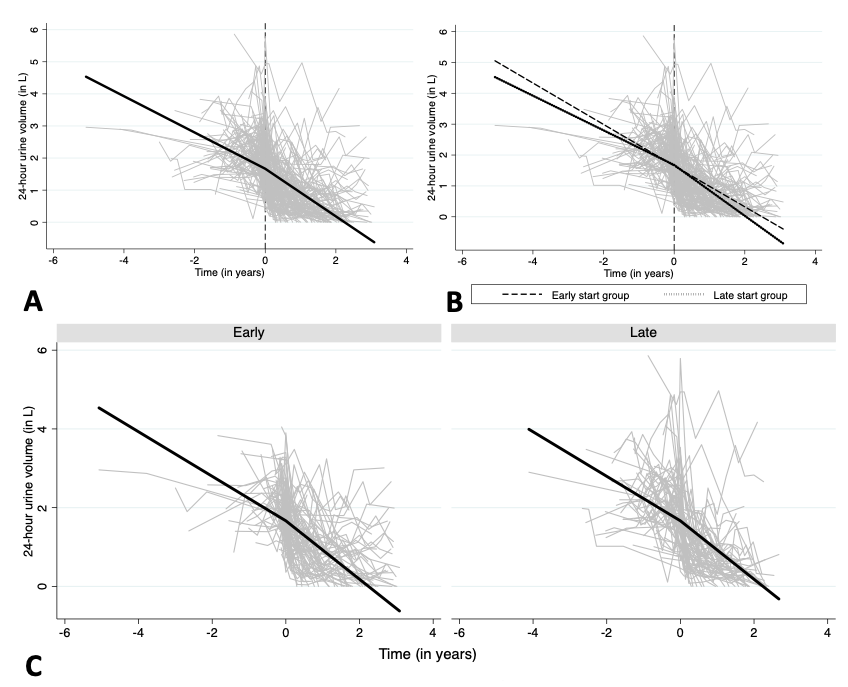

Supplement: S2 Fig — The gray lines represent individual patient measurements and the black lines represent the predicted slopes in the pre- and post-dialysis initiation periods. (TIF) [file pone.0242254.s005.tif]

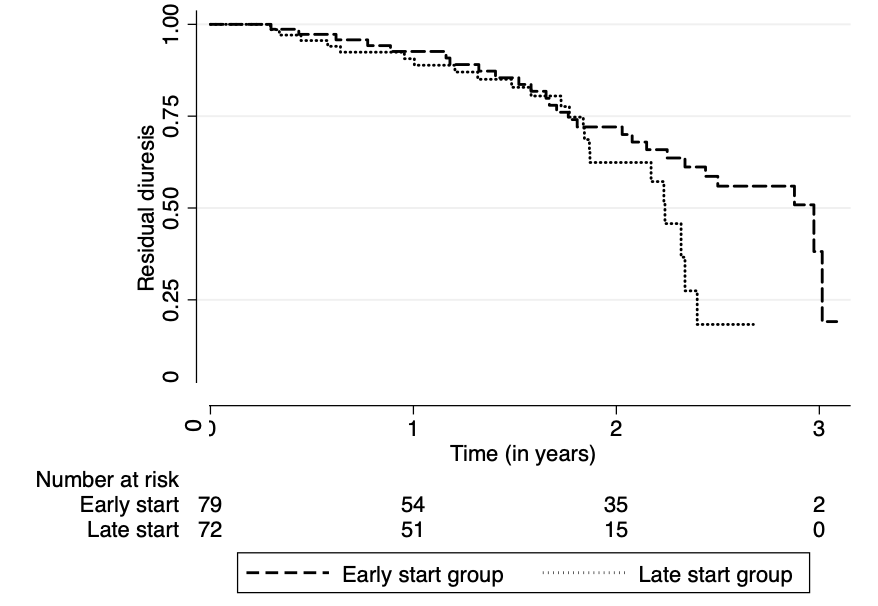

Supplement: S3 Fig — (TIF) [file pone.0242254.s006.tif]

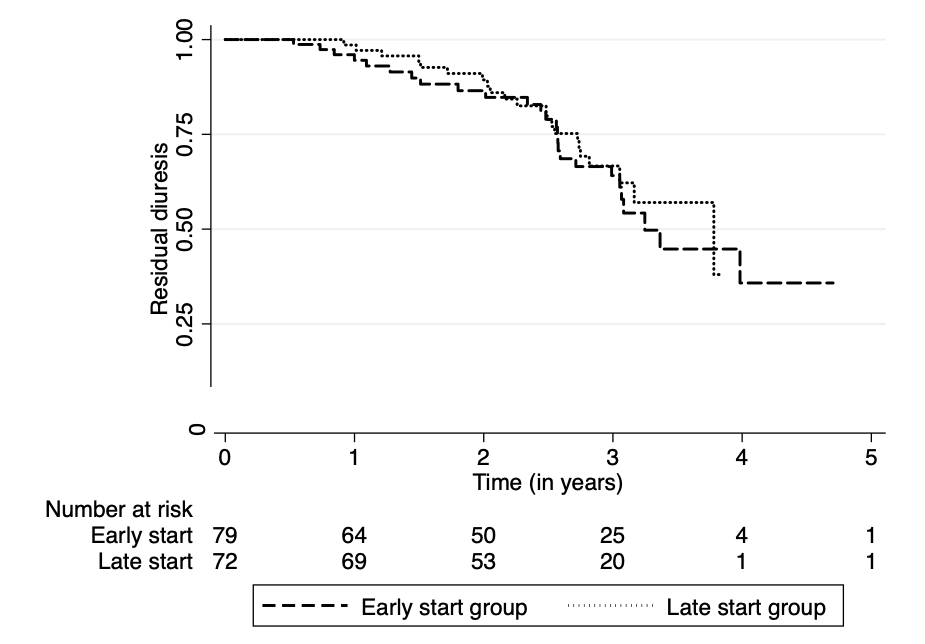

Supplement: S4 Fig — At start of observation time, GFR was similar between groups (10.3 [8.1–13.0] vs 9.9 [7.9–12.7] mL/min/1.73m2 in the early and late-start group, respectively). (TIF) [file pone.0242254.s007.tif]
