# Supplementary material for: Rate of decline in residual kidney function pre and post peritoneal dialysis initiation: A post hoc analysis of the IDEAL study
Source: PLoS One. 2020 Nov 16;15(11):e0242254. doi: 10.1371/journal.pone.0242254 (PMC7668577; doi:10.1371/journal.pone.0242254)
Supplement: S1 Study protocol — (PDF) [file pone.0242254.s009.pdf]

STUDY PROTOCOL

Rate of decline in residual kidney function pre and post peritoneal dialysis initiation: a post-hoc analysis of the IDEAL study

INVESTIGATORS

Isabelle Ethier[1]
Carmel Hawley[1]
David Johnson[1]
Yeoungjee Cho[1]
Bruce A Cooper
David C Harris
Carol A Pollock
Muh Geot Wong
[1]Department of Nephrology, Princess Alexandra Hospital, Brisbane, Australia

BACKGROUND

Residual kidney function (RKF) is associated with improved survival and quality of life in dialysis patients.[1–3] Many studies have shown that RKF is better preserved on peritoneal dialysis (PD) than on hemodialysis.[4–7] In a small retrospective observational study of 14 PD patients, Berlanga *et al.* showed a lower rate of decline of RKF during PD than during the predialysis period.[8] Similar results were found in a larger cohort of 77 new PD patients by He *et al.*[9] Results from the NECOSAD study also described an attenuation of the decline of glomerular filtration rate (GFR) from the period before the start of dialysis compared to 2 to 4 months after PD initiation.[10] In both studies previously mentioned,[9,10] a higher GFR at dialysis initiation was also an independent risk factor for RKF decline during the PD phase.

In 2010, the Initiating Dialysis Early and Late (IDEAL) study[11] randomised 828 dialysis patients to either early (GFR between 10 and 14 ml/min/1.73m^2) or late (GFR<7 ml/min/1.73m^2 or when clinical indicators of uremia supervened) start of dialysis. This study, which included 366 PD patients, showed that planned early commencement of dialysis was not associated with either a survival or a quality of life benefit compared to late initiation.

PROJECT OUTLINE

This study will examine the slope of decline in RKF in PD patients enrolled in the IDEAL study in the in the pre- and post-dialysis initiation periods.

AIMS

1. To compare RKF in the pre- and post-PD commencement periods and
2. To evaluate the impact of early vs. late initiation of PD on the decline of RKF
3. To compare time to anuria in the early vs. late initiation of PD
4. To identify the risk factors associated with anuria in patients initiating PD
5. To evaluate the association of RKF with clinical outcomes (mortality, hospitalisation)

HYPOTHESES

We hypothesise that initiating patients on PD favorably alters the rate of decline of RKF.

METHODS

This study is a *post-hoc* analysis using the datasets from the initial IDEAL trial (Australian and New Zealand Clinical Trials Registry number 12609000266268). The detailed IDEAL study design, methodology and results have been previously published.[11]

As part of the IDEAL study, adult patients with progressive chronic kidney failure with an estimated GFR (using the Cockcroft-Gault equation) between 10 and 20 ml/min/1.73m^2 were enrolled. Baseline characteristics were collected at enrollment and patients were subsequently evaluated on a 3-monthly basis, including timed 24-hour urine collection. Once GFR was first recorded to be ≤15 ml/min/1.73m^2, patients were randomised to either commence dialysis immediately ("early start") or continue routine care until dialysis initiation at GFR of 5-7 ml/min/1.73m^2 ("late start").

Only PD patients from the original IDEAL study will be evaluated for inclusion in this post-hoc analysis. PD patients will be included if results from 24-hour urine collections have been recorded within 30 days of dialysis initiation (-30 to + 30 days from start), and at least one value pre- and one value post-dialysis commencement are available. All available 24-hour urine collection results recorded during the IDEAL study, both pre- and post-dialysis start, will be reviewed as part of the present analysis.

Primary outcomes:

- Slope of decline of GFR over time, calculated as mean of creatinine and urea clearances from a 24-hour urine collection, corrected for body surface area (overall and in each treatment group)

Secondary outcomes:

- Time to anuria (time to event analysis)
- Risk factors for anuria
- Mortality and hospitalisation

Predictors / confounders:

- Patient characteristics at baseline
- Dialysis characteristics
  - Initial dialysis dose (incremental vs full)
  - Early vs late initiation

DATA COLLECTION

No new data will be collected as part of this study. All data will be obtained through the original deidentified datasets from the initial IDEAL trial.

PLANNED STATISTICAL ANALYSES

Statistical analyses will be performed by the investigators.

Continuous normally distributed variables will be compared between groups (early vs late start) with the use of Student's t-test; continuous non-normally distributed variables will be compared with the use of Mann-Whitney test; and categorical variables will be compared with the use of chi-squared test.

For the primary outcome of slope of decline in RKF over time, relevant patients' baseline characteristics will be evaluated in a univariate model. A mixed-effects linear model will then fitted be with GFR as the outcome. Time, treatment group (early vs late) and statistically significant patients' characteristics from the univariate model will be included as fixed effect covariates. Patient identification number will be used as a random intercept to account for repeated measurements over time. To allow for patient-specific intercept and slope over time, the time covariate will also be fitted as a random effect. The main model will therefore be a mixed-effect linear model with a random intercept and a random effect. GFR will be assumed to be normally distributed and to decline in a linear pattern. To allow for a change in the slope at dialysis commencement, the predicted values from the mixed model will subsequently be fitted into a piecewise regression model where a "breakdummy" will be used to identify values greater than the structural break value. Start of dialysis will be used as the break point where time will be identified as 0. This final model will estimate the rate of GFR decline per year in the pre-dialysis start period as the coefficient for time ($ß_1$) and the change in slope from the preceding interval ($ß_2$), which will enable testing for the significance of the change in slope. The rate of decline in the post-dialysis initiation period can be calculated as $ß_1 + ß_2$.

Time to event analyses (time to anuria, mortality, hospitalisations) will be performed by univariate Kaplan-Meier survival analysis and multivariate Cox proportional hazards model analyses. Multivariable logistic regression will be used to identify risk factors for anuria.

The statistical analyses will be performed using Stata (version 15.1; StataCorp LLC, Texas, USA). P<0.05 will be considered statistically significant.

ETHICAL CONSIDERATIONS & POTENTIAL FOR RISK/BURDENS

This study does not involve recruitment of patients. It does not involve a direct approach to patients and consent is not to be sought, as this is a *post-hoc* analysis from a completed study. No risk or burden is expected for the patients in this study.

The current IDEAL data is stored in a password protected database in the Renal Clinical trial unit at RNSH. The IDEAL steering committee members have overall oversight of the original IDEAL dataset. The custodian of the original IDEAL dataset remains the responsibility of the Renal Trial Unit at RNSH. Only a subset of the data designated for these substudy analyses will be shared with Princess Alexandra Hospital (PAH). Researchers will receive a de-identified set of data for the purposes of this study in which only the random code and date of birth will be available. However, consent for the re-use of identifiable data has already been obtained as part of the original IDEAL study. This new database will be shared with designated investigators at PAH Department of Nephrology. The database will be kept on a password-protected PAH registered computer in a secure location within the Princess Alexandra Hospital Department of Nephrology. PAH will assume responsibility as the data custodian for the analysis and storage of the dataset used in the present study. Access to the data will only be allowed to investigators involved in this study. The data will be kept for seven (7) years, as per Australian Code and State Archiving guidelines, after which time all data will be destroyed according to Metro South Health's policy on the destruction of digital records.

DISSEMINATION OF RESULTS AND PUBLICATIONS

Results from this study are intended to be published as an article in a peer-reviewed journal and/or abstract, poster or oral presentation during local or international scientific meetings. In this eventuality, all data will remain anonymous and patients will not be identified at any time, as only de-identified data will be accessed.

REFERENCES

1. Termorshuizen F, Korevaar JC, Dekker FW, Van Manen JG, Boeschoten EW, Krediet RT. The Relative Importance of Residual Renal Function Compared With Peritoneal Clearance for Patient Survival and Quality of Life: An Analysis of the Netherlands Cooperative Study on the Adequacy of Dialysis (NECOSAD)-2. *Am J Kidney Dis*. 2003;41(6):1293-1302. doi:10.1016/S0272-6386(03)00362-7
2. Termorshuizen F, Dekker FW, Van Manen JG, Korevaar JC, Boeschoten EW, Krediet RT. Relative Contribution of Residual Renal Function and Different Measures of Adequacy to Survival in Hemodialysis Patients: An analysis of the Netherlands Cooperative Study on the Adequacy of Dialysis (NECOSAD)-2. *J Am Soc Nephrol*. 2004;15(4):1061-1070. doi:10.1097/01.ASN.0000117976.29592.93
3. Perl J, Bargman JM. The Importance of Residual Kidney Function for Patients on Dialysis: A Critical Review. *Am J Kidney Dis*. 2009;53(6):1068-1081. doi:10.1053/j.ajkd.2009.02.012
4. Lysaght MJ, Vonesh EF, Gotch F, et al. The Influence of Dialysis Treatment Modality on the Decline of Remaining Renal Function. *ASAIO Trans*. 1991;37:598-604.
5. Moist LM, Port FK, Orzol SM, et al. Predictors of Loss of Residual Renal Function among New Dialysis Patients. *J Am Soc Nephrol*. 2000;11(3):556-564. http://jasn.asnjournals.org/content/11/3/556.full.pdf.
6. Lang SM, Bergner A, Töpfer M, Schiffl H. Preservation of residual renal function in dialysis patients: Effects of dialysis-technique-related factors. *Perit Dial Int*. 2001;21(1):52-58.
7. Jansen MAM, Hart AAM, Korevaar JC, Dekker FW, Boeschoten EW, Krediet RT. Predictors of the rate of decline of residual renal function in incident dialysis patients. *Kidney Int*. 2002;62(3):1046-1053. doi:10.1046/j.1523-1755.2002.00505.x
8. Berlanga JR, Marron B, Reyero A, Caramelo C, Ortiz A. Peritoneal Dialysis Retardation of Progression of Advanced Renal Failure. *Perit Dial Int*. 2002;22(2):239-242.
9. He L, Liu X, Li Z, et al. Rate of decline of residual kidney function before and after the start of peritoneal dialysis. *Perit Dial Int*. 2016;36(3):334-339. http://www.pdiconnect.com/content/36/3/334.full.pdf%0Ahttp://ovidsp.ovid.com/ovidweb.cgi?T=JS&PAGE=reference&D=emed18&NEWS=N&AN=610521888.
10. De Jager DJ, Halbesma N, Krediet RT, et al. Is the decline of renal function different before and after the start of dialysis? *Nephrol Dial Transplant*. 2013;28(3):698-705. doi:10.1093/ndt/gfs578
11. Cooper BA, Branley P, Bulfone L, et al. A Randomized, Controlled Trial of Early versus Late Initiation of Dialysis. *N Engl J Med*. 2010;363(7):609-619. doi:10.1056/NEJMoa1402121
